# Detection of Mild Cognitive Impairment Through Hand Motor Function Under Digital Cognitive Test: Mixed Methods Study

**DOI:** 10.2196/48777

**Published:** 2024-06-26

**Authors:** Aoyu Li, Jingwen Li, Jiali Chai, Wei Wu, Suamn Chaudhary, Juanjuan Zhao, Yan Qiang

**Affiliations:** 1 School of Software Taiyuan University of Technology Jinzhong China; 2 School of Computer Science Xijing University Xian China; 3 College of Computer Science and Technology (College of Data Science) Taiyuan University of Technology Jinzhong China; 4 Shanxi Provincial People’s Hospital Taiyuan China; 5 School of Software North University of China Taiyuan China

**Keywords:** mild cognitive impairment, movement kinetics, digital cognitive test, dual task, mobile phone

## Abstract

**Background:**

Early detection of cognitive impairment or dementia is essential to reduce the incidence of severe neurodegenerative diseases. However, currently available diagnostic tools for detecting mild cognitive impairment (MCI) or dementia are time-consuming, expensive, or not widely accessible. Hence, exploring more effective methods to assist clinicians in detecting MCI is necessary.

**Objective:**

In this study, we aimed to explore the feasibility and efficiency of assessing MCI through movement kinetics under tablet-based “drawing and dragging” tasks.

**Methods:**

We iteratively designed “drawing and dragging” tasks by conducting symposiums, programming, and interviews with stakeholders (neurologists, nurses, engineers, patients with MCI, healthy older adults, and caregivers). Subsequently, stroke patterns and movement kinetics were evaluated in healthy control and MCI groups by comparing 5 categories of features related to hand motor function (ie, time, stroke, frequency, score, and sequence). Finally, user experience with the overall cognitive screening system was investigated using structured questionnaires and unstructured interviews, and their suggestions were recorded.

**Results:**

The “drawing and dragging” tasks can detect MCI effectively, with an average accuracy of 85% (SD 2%). Using statistical comparison of movement kinetics, we discovered that the time- and score-based features are the most effective among all the features. Specifically, compared with the healthy control group, the MCI group showed a significant increase in the time they took for the hand to switch from one stroke to the next, with longer drawing times, slow dragging, and lower scores. In addition, patients with MCI had poorer decision-making strategies and visual perception of drawing sequence features, as evidenced by adding auxiliary information and losing more local details in the drawing. Feedback from user experience indicates that our system is user-friendly and facilitates screening for deficits in self-perception.

**Conclusions:**

The tablet-based MCI detection system quantitatively assesses hand motor function in older adults and further elucidates the cognitive and behavioral decline phenomenon in patients with MCI. This innovative approach serves to identify and measure digital biomarkers associated with MCI or Alzheimer dementia, enabling the monitoring of changes in patients’ executive function and visual perceptual abilities as the disease advances.

## Introduction

### Background

Mild cognitive impairment (MCI) is an intermediate stage between age-related cognitive decline and dementia, which affects the individual’s cognitive, social, and mental aspects and can lead to emotional problems that affect daily living [1]. A recent study has revealed that the incidence of MCI is 6.7% for individuals aged 60 to 64 years, 8.4% for those aged 65 to 69 years, 10.1% for those aged 70 to 74 years, 14.8% for those aged 75 to 79 years, and 25.2% for those aged 80 to 84 years [2]. Previous evidence has shown that patients with MCI develop dementia or Alzheimer dementia (AD) at a rate of approximately 10% to 15% per year [3,4], significantly higher than the incidence of dementia in the general population, which is 1% to 2% per year. The increasing prevalence of dementia, coupled with a high conversion rate, presents challenges not only for those directly impacted by the condition, including individuals, caregivers, and families, but also for society.

Despite the high risk of dementia for people with MCI and the need for early intervention [5], there are currently no drugs or other treatments to modify the clinical course or delay the onset of dementia [6]. In clinical settings, the diagnostic process for MCI could be costly, often involving expensive and sometimes invasive or time-consuming examinations. In addition, the early symptoms of MCI are subtle, making it easy for patients to attribute them to the normal aging process rather than to cognitive impairment and leading to an early stage where patients may not undergo clinical examination. Therefore, achieving an effective MCI diagnosis remains one of the most challenging tasks in geriatric psychiatry [7].

### Digital Drawing Tasks in MCI Detection

As a comprehensive activity, drawing requires various cognitive skills including orientation, selective and sustained attention, visual memory and reconstruction, visuospatial organization, and motor performance [8]. These requirements suggest that older adults may be susceptible to cognitive dysfunction, and an accurate assessment of their involvement in relevant activities might aid in identifying such disabilities [9]. With the development of portable devices and mobile computing, some researchers have attempted to improve drawing tests using digital methods to capture more information and influencing factors related to screening for MCI, such as working memory, attention allocation, cognitive flexibility, as well as visual and spatial processing. These factors play an essential role in the screening and assessment processes for MCI and help provide more comprehensive information about cognitive functioning, leading to a better understanding of cognitive health and possible problems in participants.

Recently, Müller et al [10] used a Windows Surface Pro 4 digitizer and a handheld stylus pen to evaluate the digital Clock Drawing Test (dCDT) and found a significant difference in time in air between the healthy older adults and the patient group. Afterward, Dion et al [11] discovered a difference in the “thinking time” percentage between the groups. The MCI group took 10 seconds longer to draw their clocks from memory, suggesting that the difference between those with and without MCI may lie in grouping and coordinating the necessary cognitive resources rather than severe drawing errors. Another study demonstrated differences in the time to completion, total pen stroke count, and higher-order decision-making latencies of dCDT between age groups, involving participants recruited as free of dementia and stroke [12]. As they transition from one part of the drawing to the next (eg, postclock delay), participants may use more diverse neurocognitive resources than simply processing speed. In addition to assessing the Clock Drawing Test (CDT), other drawing tasks were also studied. Kim et al [13] created a simplified Rey-Osterrieth Complex Figure Test (sRCFT) to assess digital pen strokes, spatial arrangement, and similarity of drawings in the drawing process, finding that these measures could serve as valuable digital biomarkers for studying visual structural dysfunction in AD. In addition, Müller et al [14] investigated the movement kinematics (ie, time in air, time on surface, and total time) of older adults with MCI when copying a 3D house to reflect their manual dexterity, visual space construction, and other cognitive abilities. Finally, in the digital tree drawing test, patients with MCI and early-stage AD used fewer colors and line widths, and their images displayed reduced contrast and heterogeneity [15].

Digital drawing tasks provide standardized test administration, more detailed feedback metrics, and automated scoring than traditional paper-based tests. However, these assessment tasks typically focus on the patient’s overall performance, neglecting to capture detailed features (ie, clock face, numbers, and pointers in dCDT). In modern digital devices, tracking subtle cognitive changes in patients’ selection of drawing areas, organization of shapes, and determining image sequences may offer a more practical approach to MCI detection.

### Dual-Task Paradigm in MCI Detection

The dual-task paradigm is an experimental design approach for exploring the effects of multitasking on cognitive performance and brain function. In the dual-task paradigm, participants must perform ≥2 tasks simultaneously, involving different cognitive processes, such as attention, stimulus encoding, decision-making, working memory, response selection, and execution. This paradigm could detect changes in dual-task performance early in the progression of the most common neurodegenerative diseases [16]. Compared to other cognitive assessment tools, the dual-task paradigm is less influenced by the education level and is more applicable to participants with different educational backgrounds. In addition, the dual-task paradigm is quick, practical, and easy to apply in clinical practice, making it a promising method for cognitive assessment [17].

In studies related to the diagnosis of MCI, the analysis of dual-task gait tests has proven to be a valid method. The dual-task gait test is a commonly used clinical assessment that requires participants to perform a cognitive task while simultaneously walking. In other words, participants must complete additional cognitive tasks such as counting, recalling words, or performing attention shifts while walking. For example, Montero-Odasso et al [18] conducted a dual-task gait test on 112 older adults with MCI and followed them for 6 years. Interestingly, high dual-task gait costs were associated with a 3.8-fold and a 2.4-fold increased risk of progression to dementia when counting backward and naming animals, respectively. Whitson et al [16] investigated gait-cognitive dual-task performance in 29 older adults and found that APOE ɛ4 carriers exhibited more pronounced dual-task interference than low-risk participants. Compared to electronic sidewalks, Aoki et al [19] used a Kinect sensor to capture the whole-body movements of participants and extracted more substantial gait feature information by improving the motion performance capture method. Their findings revealed that a classifier based on dual-task gait features could detect older adults who scored low on the Mini-Mental State Examination (MMSE) test [20]. In addition, Ali et al [21] used the Vicon Nexus 2.8 motion capture system to obtain kinematic gait parameters such as velocity, peak knee extension angle, and dual-task cost for participants. Their study showed that the kinematic gait parameters of the dual-task peak knee extension angle for story recall gait could effectively differentiate the MCI group from the healthy control (HC) group.

Currently, methods for detecting MCI using the dual-task paradigm focus on gait-motor assessment or developing new screening tools. Commonly used screening methods for cognitive impairment in older adults (ie, CDT and Rey-Osterrieth Complex Figure Test [RCFT]) by neurologists, psychiatrists, and general practitioners still have good clinical outcomes due to their rapid administration, patient acceptability, and simple scoring rules [22,23]. Thus, integrating a dual-task paradigm into clinical screening tools may provide more alternatives to MCI detection.

### Objective

We designed tablet-based “drawing and dragging” tasks, prototyped with clinically validated drawing tests, and explored the feasibility and efficiency of assessing MCI through movement kinetics under these tasks. In this study, the drawing task improves the traditional test clinicians use to fulfill the requirements for detecting richer cognitive domains. However, the dragging task is designed to meet the cognitive screening of older adults alone at home by providing a broader and more convenient means of self-examination and early warning. Finally, we hypothesized that movement kinetics features extracted from participants during drawing or dragging could effectively distinguish patients with MCI from healthy older adults.

## Methods

### Ethical Considerations

This research was reviewed and approved by the Biomedical Ethics Review Committee of Taiyuan University of Technology (20230118). The patients and participants provided their written informed consent to participate in this study. We provided US $10 to eligible older adults as compensation for participation.

### Experimental Design

#### Design Overview

**Figure 1:**
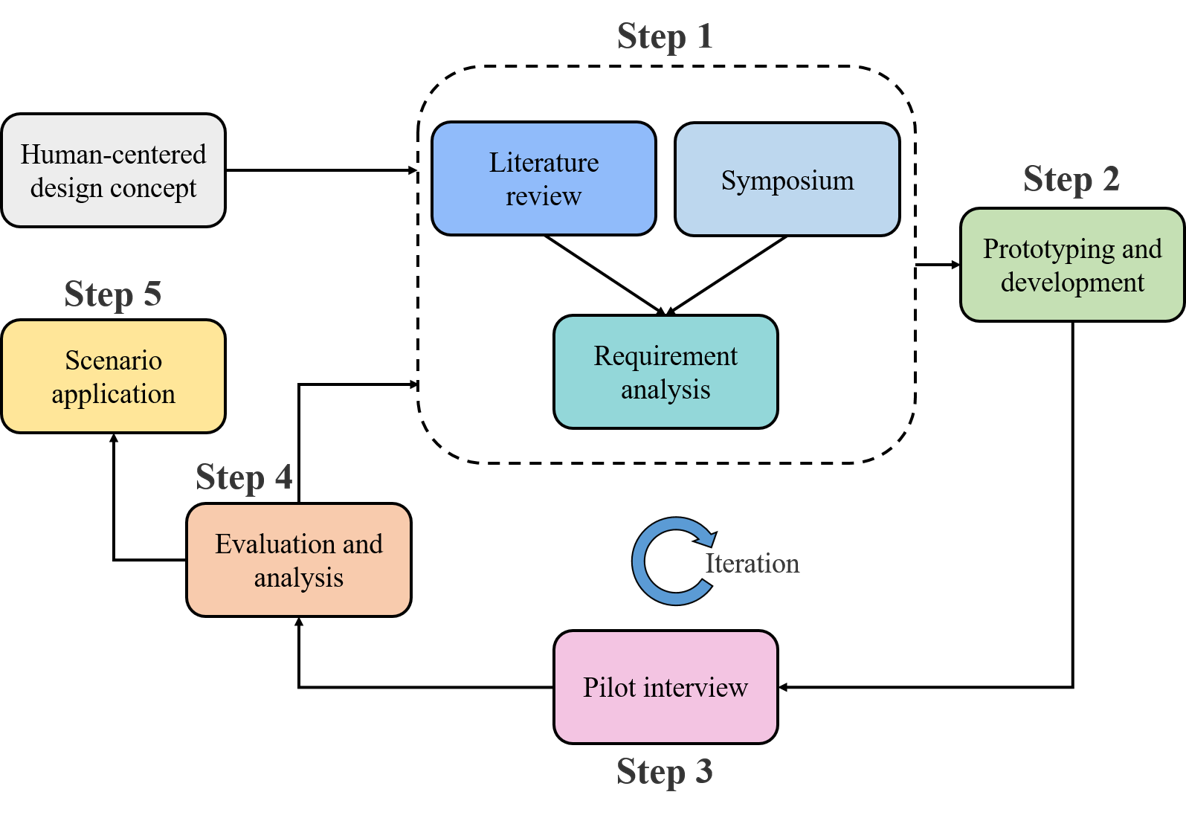
The human-centered design approach is applied to the process of cognitive tasks, and the first 3 steps form an iterative design circle to improve and adapt the tasks continuously.

To design simple and effective “drawing and dragging” tasks for assessing hand motor function, we applied a human-centered design approach [24], as shown in Figure 1. Specifically, the approach included (1) a literature review and symposium to analyze the requirements of patients with MCI, (2) prototype design and development, (3) pilot interviews, (4) system evaluation and analysis, and (5) scenario application. In step 1, we surveyed approximately 100 previous studies and performed a detailed analysis of relevant studies. In addition, we conducted symposiums, mainly involving communication between patients with MCI, healthy older adults, caregivers, neurologists, nurses, and engineers regarding what to do and how to interact. These efforts were mainly aimed at extracting user requirements and applying them to our cognitive system. On the basis of these findings, the engineers designed and developed a task prototype in step 2. Then, in step 3, we conducted pilot interviews to better understand the different preferences and demands of patients with MCI. In other words, the interviews were semistructured exchanges, and the experimental prototype was tested among healthy older adults, patients with MCI, and caregivers to refine our task further. These first 3 steps constitute an iterative design circle to continuously improve and adapt the task to the requirements of patients with MCI. In step 4, we evaluated the extent to which the designed task could effectively differentiate between patients with MCI and HC participants and how well the user experience was. Step 5 is the scenario application, that is, the extension to home, community, and clinical settings. In the experimental design phase, the main focus is on steps 1, 2, and 3 and the iterations between them, while the evaluation and analysis (step 4) are described in the *Results* section.

#### Stakeholders

The stakeholders engaged in the experimental design encompass our development team (2 neurologists, 2 nurses, and 3 engineers), patients with MCI, healthy older adults, and caregivers. Neurologists and nurses are from our partner hospitals and have been working for 10 years on diagnosing and treating neurological diseases. The engineers comprised 1 PhD and 2 MS students specializing in human-computer interaction. In addition, we recruited 49 patients with MCI, 49 healthy older adults, and 4 caregivers. They were mainly from the community, nursing home, and hospital. All older adults completed the Montreal Cognitive Assessment (MoCA) test [25], the Clinical Dementia Rating (CDR) test [26], and an informed consent form and underwent comprehensive medical evaluations conducted by experienced neurologists (including detailed medical history, systematic physical examination, and imaging studies). Furthermore, neurologists conducted clinical interviews with patients or their caregivers and collected self-reports to assess and diagnose MCI comprehensively. Among the 49 patients with MCI, all had MoCA scores <26 and CDR scores of 0.5. Brain magnetic resonance imaging or computed tomography scans revealed no other structural abnormalities associated with cognitive impairment. We provided the participants with a notebook and a pen

#### Prototype 1: Design and Iteration

Using clinically validated paper-based drawing tasks as screening elements, we conducted a comprehensive review of clinical MCI screening tools and invited 4 (8%) of the 49 patients with MCI and 4 (8%) of the 49 healthy older adults to participate in a symposium. We focused on the following points:

1. Which of the existing MCI diagnostic methods in the clinical setting involve hand motor function?
2. Does the screening tool address integrated cognitive abilities (ie, memory, attention, visuospatial, motor planning, and executive functions)?
3. Does the application of screening tools extend to hospitals, nursing homes, or households?
4. The psychology of older adults, considering their preferences and cognitive limitations

The symposium results identified the CDT and RCFT as the primary tasks for the cognitive assessment system. Subsequently, in step 2 (Figure 1), the engineers designed and developed prototype 1 based on these tasks. Prototype 1 consisted of a tablet-based dCDT and a digital RCFT. The dCDT required participants to complete three tasks in the clock drawing area: (1) draw a clock face, (2) write all the numbers in the correct position, and (3) use the pointers to indicate “10 minutes past 11-o’clock.” Similarly, in the digital RCFT, participants were prompted to copy the Rey-Osterrieth Complex Figure [ROCF] on the left side within the copy area. Next, to assess the feasibility of the 2 drawing tasks in prototype 1 among older adults, we conducted interviews (step 3; Figure 1) with 6 older adults (patients with MCI: 3/49, 6%; healthy older adults: 3/49, 6%) from the community. The interviews involved engaging the older adults in the tests and soliciting their feedback and opinions. Some common dialogues from the study participants are as follows:

Experimenter: Could you share your thoughts on these tests?

Patient with MCI: What time do the pointers indicate? I forgot.

Patient with MCI: Do others have their minute pointers pointing at the number ‘2’? Am I the only one with the minute pointer pointing at the number ‘10’?

Patient with MCI: This figure (RCFT) is too complex to complete, and I prefer not to continue.

Healthy individual: Am I performing as well as others?

Healthy individual: Does it matter that the number of positions are painted at different intervals?

Healthy individual: RCFT is too time-consuming, and my eyes are tired.

#### Prototype 2: Design and Iteration

**Figure 2:**
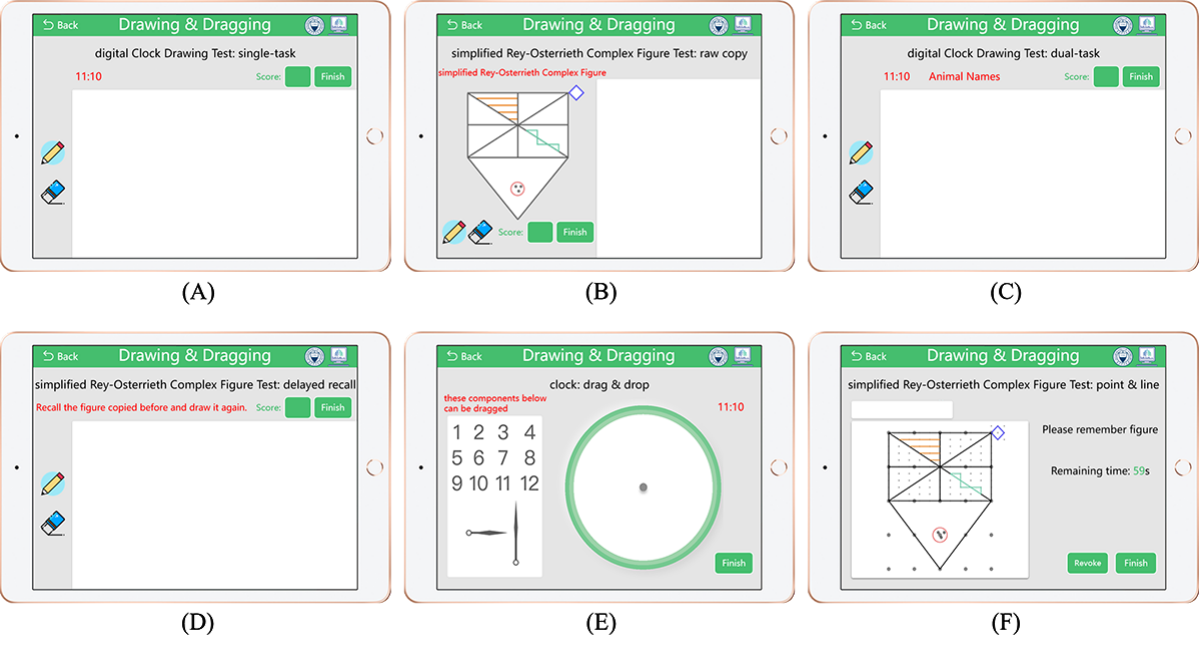
Interactive interface of drawing and dragging tasks. (A) digital Clock Drawing Test (dCDT; single task), (B) simplified Rey-Osterrieth Complex Figure Test (sRCFT; raw copy), (C) dCDT (dual task), (D) sRCFT (delayed recall), (E) clock “drag and drop”, (F) sRCFT “point and line”.

**Figure 3:**
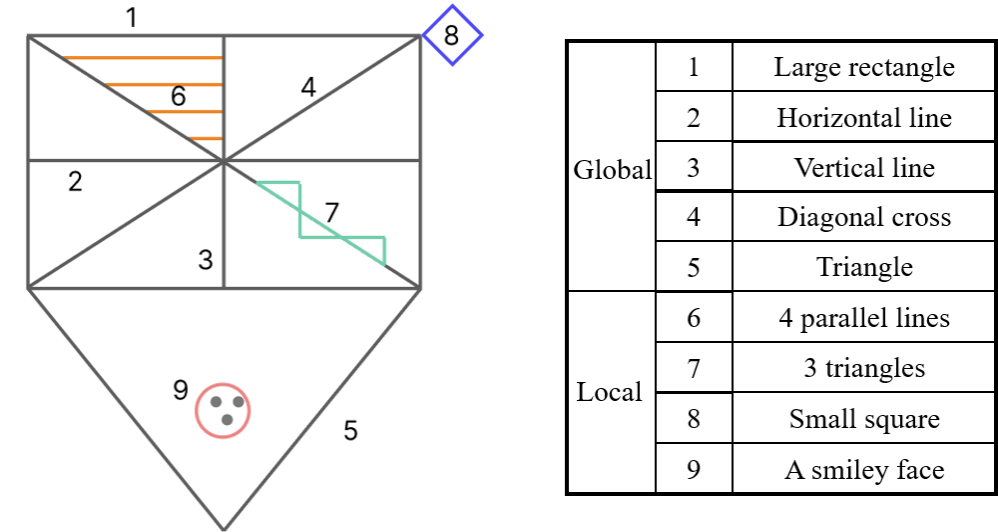
The simplified Rey-Osterrieth Complex Figure consists of 5 global and 4 local components.

Using the pilot study (step 3; Figure 1), we found that older adults were somewhat confused by the rules of dCDT (Figure 2A) but were generally receptive and engaged in its implementation. However, the digital RCFT was less than ideal. Participants thought that the ROCF was too complex and challenging to complete at first glance. In addition, it typically took ≥10 minutes to complete, which was not user-friendly to older adults. Hence, in the second symposium, we modified the original figure to a relatively simplified version while retaining its main framework. The modification work was collaboratively undertaken by 2 neurologists from our affiliated hospital, each with >10 years of experience, to ensure a balanced representation of global and local components. While the original RCFT comprises 18 components (4 global and 14 local components) [27], our simplified version of the RCFT consists of 5 global components and 4 local components, as illustrated in Figure 3.

In the global components section, we maintained the large rectangle with horizontal, vertical, and diagonal crossings; the large triangle (only the position has changed); and the 4 horizontal lines in the upper left panel, while the rest of the regions or local components have been simplified. Regarding the local components section, we made the following modifications: (1) squares replaced the diamonds, (2) the 3-point circle was moved to the bottom, and (3) three triangles staggered up and down instead of 5 parallel lines. We staggered the triangles up and down to allow participants to focus more on local components. Finally, some overlapping lines and detail parts outside the outline were removed in the simplified version. After the development, a raw copy of sRCFT is shown in Figure 2B.

**Figure 4:**
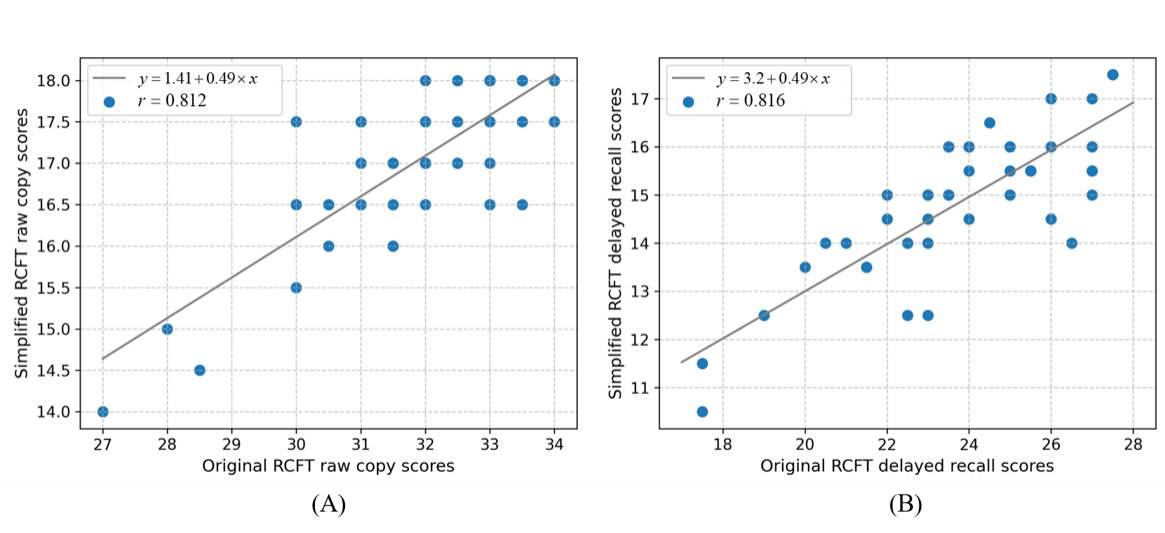
Correlation between raw copy and delayed recall scores on original and simplified RCFT: (A) raw copy and (B) delayed recall. RCFT: Rey-Osterrieth Complex Figure Test.

Subsequently, we conducted another pilot interview to investigate the performance of the sRCFT compared to the original RCFT on cognitive screening. We recorded the distribution of original RCFT and sRCFT scores for 24 (49%) out of 49 healthy older adults and 24 (49%) out of 49 patients with MCI in the raw copy and delayed recall (waiting 20 minutes to recall and draw the figure) tasks. The normality test for the score variables revealed that none of the groups met normality. Therefore, we used the median (IQR) for statistical description and the nonparametric Mann-Whitney *U* test for comparison between groups. As depicted in Table 1, the HC group scored significantly higher than the MCI group on the delayed recall task, while no significant difference was observed between the 2 groups in the raw copy task. Moreover, we examined the correlation between the raw copy and delayed recall scores of the original RCFT and sRCFT using Spearman correlation analysis. A positive correlation was noted between the original RCFT and sRCFT score (raw copy: *r*=0.812; *P*<.001 and delayed recall: *r*=0.816; *P*<.001; Figure 4).

**Table 1:** Raw copy and delayed recall scores of healthy older adults and patients with mild cognitive impairment on the original and simplified Rey-Osterrieth Complex Figure Test (RCFT).

	HC^a^ group (n=24)	MCI^b^ group (n=24)	*P* value	*z* score
Original RCFT: raw copy (IQR 0-36)	32.5 (31.5-33.5)	32 (31-33)	.18	−1.350
Simplified RCFT: raw copy (IQR 0-18)	17.25 (16.5-17.5)	17 (16.5-18)	.94	−0.074
Original RCFT: delayed recall (IQR 0-36)	25.25 (24-27)	22.75 (21-24.5)	<.001	−3.639
Simplified RCFT: delayed recall (IQR 0-18)	16 (15-16.875)	14 (13.5-15)	<.001	−4.208

^a^HC: healthy control.

^b^MCI: mild cognitive impairment.

#### Prototype 3: Design and Iteration

As is well known, MCI encompasses changes in cognitive performance, including declines in short-term memory, working memory, logical thinking, verbal expression, spatial cognition, and executive function [24]. To comprehensively assess these aspects, we enhanced the drawing task during the third symposium, which involved adding 3 dual tasks to the dCDT and introducing a 10-minute delayed recall task in the sRCFT, as depicted in Figures 2C and 2D. In this prototype, 3 language function–related tasks include series 1 (low load: count backward from 100), animal names (medium load: name as many animals as possible), and series 3 (high load: subtract 3 from 100 and give the result) [28,29]. We combined the 3 language function–related tasks with the dCDT, resulting in 3 dual tasks. In the dual task, participants were required to perform a single dCDT task while completing a task related to language function. Series 1 was applied to test essential language functions, whereas series 2 and 3 focused on memory and logical thinking skills, respectively. Finally, according to the Schulman criteria, the dCDT score ranges from 1 point (ie, a perfectly accomplished CDT) to 6 points (ie, severe impairment and no identifiable clock) [30].

Performance in the delayed recall condition helps examiners assess visuospatial memory in declarative memory associated with the hippocampus and related areas of the right temporal lobe [31,32]. However, in the traditional delayed recall task, participants were asked to recall the previous figure 30 minutes after completing the RCFT raw copy task. Long waits are unwelcome and unpleasant for older adults. Therefore, we shortened the delayed recall time to 10 minutes [33] and adjusted the drawing sequence. The participants’ performance in sRCFT will be scored in accuracy and location [34]. The score weights of the 9 components are equal, namely 2 (accurately drawn and correctly located); 1 (accurately drawn and incorrectly located or inaccurately drawn and correctly located); 0.5 (inaccurately drawn and incorrectly placed but identifiable); or 0 (inaccurately drawn, incorrectly located, and unidentifiable). Therefore, the possible range of raw scores is 0 to 18. A lower score indicates a more severe visual perception or construction function impairment.

In addition, during the nursing home interviews, 4 (8%) out of 49 healthy older adults mentioned that they were not accustomed to using the Apple Pencil for drawing and suggested incorporating a practice mode before initiating the test. Following discussions with the development engineers, we promptly integrated their feedback and added a practice mode, allowing free drawing without a time limit. At the same time, we assisted in teaching during the practice process to ensure that the participants could use the Apple Pencil in their experiments.

#### Prototype 4: Design and Iteration

To meet the demands for cognitive screening for older adults alone at home, we designed “drag and drop” and “point and line” tasks to indirectly assess their cognitive status by capturing subtle changes in hand motor function. Participating experimental designers included 4 (8%) out of 49 patients with MCI, 4 (8%) out of 49 healthy older adults, 2 (50%) out of 4 caregivers, and all our team members.

Similar to drawing, drag and drop is used to assess hand motor function and detect potential cognitive decline in patients with MCI [35,36]. So, we designed a clock “drag and drop” task, as shown in Figure 2E. The clock “drag and drop” task requires participants to drag or drop numbers and pointers from the drag area to the inside of the clock face, then rotate the pointers to point “10 minutes past 11.” On the other hand, concerning sRCFT, we transformed the drawing task into a “point and line” task, as shown in Figure 2F. First, the participants were asked to memorize the simplified ROCF (including his position, belt, and all details) for 60 seconds. Then, the participant began recalling, connecting dots, and drawing lines to reproduce the target pattern. Specifically, the clock “drag and drop” task was scored on a scale of 0 to 3 (ie, numbers position, numbers order, and pointers indication; a high score indicates a perfect clock). The sRCFT “point and line” task has the same scoring rules as the sRCFT drawing task (ie, a score between 0 and 18). In addition, during the pilot interviews, 4 (8%) out of 49 patients with MCI and 4 (8%) out of 49 healthy older adults from our partner hospitals completed the tests.

### Experimental Participants and Procedure

Before initiating the study, we conducted a power analysis to estimate the sample size required to detect significant differences between the MCI and HC groups. This analysis considered an expected effect size of 0.3, a significance level of .05, and a statistical power of 0.8, requiring at least 82 participants per group [37]. Similar research in the field also informed our sample size, which typically used participant numbers within this range for comparable outcomes [38-41].

We rerecruited 207 participants from the geriatrics and neurology department research clinics at our collaborating hospitals who had not previously been involved in the design or development of the experiment. Participants were identified using a purposive sampling method [42], with the process being meticulously overseen by experienced neurologists. The inclusion criteria were that participants must (1) have normal hearing and vision or corrected to normal, (2) be aged >65 years, (3) have completed the MMSE test, (4) have completed the MoCA test, (5) have completed the CDR test, (6) be capable of moderate exercise without physical disabilities, (7) have no severe depressive symptoms or other mental illnesses, (8) be capable of using smart devices (eg, smartphones and tablets). Neurologists contacted potential participants during their clinic visits and explained the study’s purpose, related procedures, and the possible impact of the research findings. Once potential participants expressed interest, neurologists conducted comprehensive medical evaluations, including detailed medical history collection; physical examinations; brain imaging (magnetic resonance imaging or computed tomography scans); and cognitive function assessments (using the MMSE, MoCA, and CDR scales). Of 207 participants, the MCI group comprised 108 (52.2%) participants who scored <26 on the MoCA and had a CDR score of 0.5, while the HC group included 99 (47.8%) healthy older adults without symptoms of cognitive decline. Brain imaging scans revealed no structural abnormalities causing cognitive impairment. Furthermore, all patients with MCI met the criteria proposed by the National Institute of Neurological and Communicative Disorders and Stroke and the Alzheimer’s Disease and Related Disorders Association [43]. We also administered the habitual hand questionnaire [44], which consisted of 13 items, to all participants. The MCI and healthy groups were matched for age, gender, hand preference, education, average sleep duration (in general), exercise habit (regularly engaging in exercise or infrequently), and years of smart device use. Table 2 summarizes the clinical and demographic information for all participants.

**Table 2:** Clinical and demographic characteristics (n=207).

		MCI^a^ (n=108)	HC^b^ (n=99)	*P* value	
Age (y), mean (SD)		71.34 (4.48)	70.11 (4.00)	.13	
**Gender, n (%)**					.81
	Woman	64 (59.2)	57 (57.6)		
	Man	44 (40.7)	42 (42.4)		
**Hand preference, n (%)**					.43
	Left	11 (10.2)	7 (7.1)		
	Right	97 (89.8)	92 (92.9)		
Education years, mean (SD)		6.21 (3.28)	6.81 (2.89)	.31	
Hours of sleep, mean (SD)		5.93 (1.20)	6.28 (1.06)	.11	
**Exercise habit, n (%)**					.63
	Yes	52 (48.1)	51 (51.5)		
	No	56 (51.9)	48 (48.5)		
Smart device use years, mean (SD)		5.52 (2.54)	5.87 (2.52)	.47	
MoCA^c^, mean (SD)		24.09 (1.13)	27.04 (1.21)	<.001	
MMSE^d^, mean (SD)		25.31 (1.13)	28.00 (0.92)	<.001	

^a^MCI: mild cognitive impairment.

^b^HC: healthy control.

^c^MoCA: Montreal Cognitive Assessment.

^d^MMSE: Mini-Mental State Examination.

We implemented digital “drawing and dragging” tasks on the Lenovo Qitian M530-A154 (AMD Ryzen 7 PRO 2700/16 GB). Our system introduction video can be found in Multimedia Appendix 1. Before the experiment began, participants could draw freely with an Apple Pencil until they were prepared to collect data. After the practice mode, given the sRCFT (delayed recall) task rules, we clarified the execution sequence of digital drawing tasks, namely (1) sRCFT raw copy, (2) dCDT single task, (3) dCDT dual task, (4) sRCFT delayed recall, (5) clock “drag and drop,” and (6) sRCFT “point and line.” All tests were run on an iPad 2019 tablet (seventh generation, 3 GB/128 GB, with 10.2 inches and 2160×1620 touch screen), and Apple Pencil was configured for drawing and lining. The experimenter is engaged in the entire data collection process, clarifying the experiment’s rules and procedures for the participants and documenting their feedback. At the beginning of the experiment, the experimenter explained the whole process to the participants and encouraged them to draw freely to familiarize themselves with the use of Apple Pencil. Once participants felt prepared, the test commenced.

### Structured Questionnaire and Unstructured Interview

A structured questionnaire was used to investigate users’ experiences of the overall cognitive screening process. The questionnaire is rated on a scale of 1 to 5, with higher scores indicating better performance in this area. The questionnaire consisted of four questions.

- Question 1: are the tasks easy to understand and interact with?
- Question 2: do the tasks evoke self-awareness and inspire them?
- Question 3: are the tasks are interesting?
- Question 4: would the tasks be used consistently in future daily life?

The unstructured interview was designed to gather user feedback on the digital cognitive tests, aiming to enhance the system further. The discussion centered on two primary themes:

(1) what are your thoughts on the “drawing and dragging” tasks? (2) What aspects do you believe require optimization?

### Data Processing and Feature Extraction

Tablet-based “drawing and dragging” tasks can assess the hand motor function and cognitive abilities of older adults. The data obtained from these tests were used for feature extraction, serving as a digital biomarker to distinguish individuals with MCI from those who were healthy. Therefore, we conducted data cleaning procedures before extracting the features, which involved removing outliers and ensuring data consistency. Next, we extracted 5 features related to hand motor function, including time, stroke, frequency, score, and sequence. All these features were extracted from the drawing or dragging tasks participated by older adults. A comprehensive overview of the details of these 5 categories of features can be found in Multimedia Appendix 2.

### Statistical Analysis

Statistical analysis was conducted using SPSS software (IBM Corp) to analyze tablet device demographic characteristics and cognitive data. Age, years of education, sleep duration, years of smart device use, MoCA scores, and MMSE scores were described using means (SDs), while gender, hand preference, and exercise habits were presented as percentages. Furthermore, we conducted the Kolmogorov-Smirnov test to assess the normal distribution of all variables. For those variables conforming to a normal distribution, we applied 2-tailed *t* tests (for continuous variables) and chi-square tests (for categorical variables) to determine the significance of intergroup differences. For nonnormally distributed variables, we used the nonparametric Mann-Whitney *U* test to assess intergroup differences and estimated CIs using the Hodges-Lehmann estimator. In addition, logistic regression analysis was performed to evaluate the diagnostic value of the chosen variables in discriminating between healthy older adults and patients with MCI. The statistical significance level for all tests was set at *P*<.05.

## Results

### Analysis of Hand Motor Function Features

Table 3 exhibits the statistical comparison results between the MCI and HC groups concerning hand motor function features. In terms of MCI detection, the findings reveal discrepancies in the features selected across different tasks, indicating variations in the number of valid features among them. For instance, in the dCDT tasks (ie, dCDT single task, dCDT series 1, dCDT animal names, and dCDT series 3), the number of features with significant (*P*<.05) was 7, 7, 2, and 8, respectively. Furthermore, our analysis based on the number of selected features indicates that time- and score-based features outperformed stroke- and frequency-based features. Notably, features with significant (*P*<.001) were predominantly observed in time- and score-based features, while they were rare in stroke- and frequency-based features (only observed in sRCFT raw copy and clock drag and drop). Particularly, significant differences between the 2 groups were observed regarding time in the air, time on the surface, and time being dragged. Compared to the MCI group, the HC group exhibited significantly shorter times for switching between strokes and quicker drawing or dragging speeds. Similarly, the *P* values of the score features were mostly <.05, except for the sRCFT task (raw copy), which yielded the preferred features for subsequent logistic regression model construction. However, the dCDT animal names outcome was deemed unsatisfactory. During data collection, it was observed that most participants (151/207, 72.9%) were more accustomed to using the names of the 12 Chinese zodiac animals (Chinese folk culture), which may coincide with the clock numbers (1-12).

**Table 3:** Statistical comparisons were conducted for 4 categories of features between healthy older adults and patients with mild cognitive impairment.

Task	Time, *P* values								Stroke, *P* values							Frequency, *P* values				Score, *P* values
	1^a^	2^b^	3^c^	4^d^	5^e^	6^f^	7^g^	8^h^		9^i^	10^j^	11^k^	12^l^	13^m^	14^n^		15^o^	16^p^	17^q^	
T1^r^	.233	.525	<.001	.152	.061	<.001	<.001	.033		.121	.261	.239	.014	.077	.237		.024	.274	<.001	
T2^s^	.730	.235	.013	.265	.451	<.001	.256	.017		.065	.057	.632	.155	.011	.489		.045	.006	<.001	
T3^t^	.625	.156	.071	.059	.077	.022	.366	.641		.095	.115	.541	.514	.513	.093		.336	.532	.019	
T4^u^	.254	.785	<.001	.082	.084	<.001	.514	.012		.025	.074	.365	.224	0.002	.258		.009	.008	<.001	
T5^v^	.299	—^w^	<.001	—	—	<.001	—	—		.054	—	—	.088	—	—		<.001	—	.569	
T6^x^	<.001	—	<.001	—	—	<.001	—	—		.006	—	—	.067	—	—		.399	—	<.001	
T7^y^	.058	<.001	—	<.001	—	—	—	—		—	—	—	—	—	<.001		—	—	.029	
T8^z^	<.001	—	—		.457	—	<.001	—		—	—	—	—	—	—		—	.004	.006	

^a^1: thinking time for the first stroke.

^b^2: circle painting time or umber drag time.

^c^3: number painting time or figure painting time.

^d^4: pointer painting time or pointer drag time.

^e^5: circle unpainted time or global drawing time.

^f^6: number unpainted time or figure unpainted time.

^g^7: pointer unpainted time or local drawing time.

^h^8: circle total stroke.

^i^9: number total stroke or figure total stroke.

^j^10: pointer total stroke.

^k^11: circle pen-up stroke.

^l^12: number pen-up stroke or figure pen-up stroke.

^m^13: pointer pen-up stroke.

^n^14: circle painting frequency or hand drag frequency.

^o^15: number painting frequency or figure painting frequency.

^p^16: pointer painting frequency or figure drawing frequency.

^q^17: score.

^r^T1: digital Clock Drawing Test single task.

^s^T2: digital Clock Drawing Test dual task (series 1).

^t^T3: digital Clock Drawing Test dual task (animal names).

^u^T4: digital Clock Drawing Test dual task (series 3).

^v^T5: simplified Rey-Osterrieth Complex Figure Test raw copy.

^w^Not applicable.

^x^T6: simplified Rey-Osterrieth Complex Figure Test delayed recall.

^y^T7: clock drag and drop.

^z^T8: sRCFT point and line.

### Analysis of Drawing Sequences

We converted the drawing of the dCDT and sRCFT into pseudocolor images encoded by a series of colors in the order of strokes (Multimedia Appendix 3). In the dCDT single task, patients with MCI tended to rotate the tablet when writing numbers (making some numbers look upside down) and drawing lines to determine the 12 o’clock direction (MCI group: numbers 2 and 5 in Multimedia Appendix 3). Furthermore, upon analyzing the dCDT dual task, we noted that healthy older adults may exhibit errors in number positioning and slight gaps between numbers in series 1s due to increased cognitive load (HC group: numbers 2, 3, and 4 in Multimedia Appendix 3). As anticipated, both groups encountered challenges with the relatively complex series 3s. Notably, even some healthy older adults displayed significant gaps between numbers. However, in the spatial organization of the sRCFT, there was no discernible difference between the 2 groups (ie, global-first or local-first approach or top-first and bottom-second strategies). Particularly, healthy older adults exhibited good recall of figures, albeit with minor omissions in some details. Conversely, patients with MCI showed poor recall of figures, indicating deficits in visual memory and spatial construction abilities.

### Diagnostic Value of “Drawing and Dragging” Tasks

To further explore the diagnostic value of the designed tasks in distinguishing patients with MCI from HC participants, we used a forward stepwise inclusion method in which the features extracted from the task were entered into a logistic regression model, where the diagnostic group (HC vs MCI) was considered as the dependent variable, and the task-extracted features were used as the independent variables, as shown in Table 4.

**Table 4:** Digital drawing and dragging tasks to construct logistic regression models with selected variables and their performance.

Model and selected variable		β	OR^a^ (95% CI)	*P* value
**dCDT^b^: single task**				
	Score	1.430	4.180 (2.070-8.441)	<.001
	Number unpainted time	0.178	1.195 (1.075-1.327)	.001
	Pointer unpainted time	0.326	1.385 (1.166-1.646)	<.001
**dCDT: dual task**				
	Number painting time	0.415	1.515 (1.228-1.869)	<.001
	Number unpainted time	0.092	1.097 (1.036-1.161)	.001
	Number painting frequency	0.343	1.409 (1.080-1.837)	.01
**sRCFT^c^: raw copy**				
	Figure painting time	0.141	1.152 (1.071-1.238)	<.001
	Figure unpainted time	0.168	1.183 (1.085-1.290)	<.001
**sRCFT: delayed recall**				
	Score	−0.689	0.502 (0.339-0.744)	.001
	Figure painting time	0.149	1.161 (1.058-1.274)	.002
	Figure unpainted time	0.096	1.101 (1.028-1.178)	.006
**C** **lock: drag and drop**				
	Number drag time	0.135	1.145 (1.066-1.230)	<.001
	Pointer drag time	0.260	1.297 (1.147-1.468)	<.001
	Hand drag frequency	0.487	1.627 (1.268-2.087)	<.001
**sRCFT: point and line**				
	Thinking time for the first stroke	−0.856	0.425 (0.259-0.696)	.001
	Local drawing time	0.220	1.246 (1.032-1.506)	.02
	Local components score	0.118	1.126 (1.063-1.192)	<.001

^a^OR: odds ratio.

^b^dCDT: digital Clock Drawing Test.

^c^sRCFT: simplified Rey-Osterrieth Complex Figure Test.

**Figure 5:**
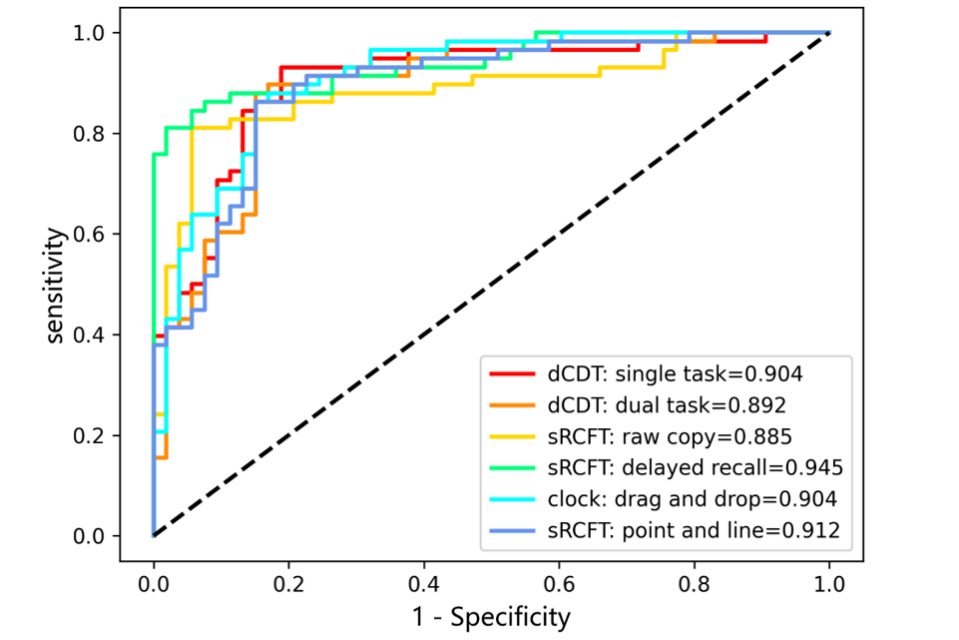
Receiver operating characteristic curves of our cognitive system. dCDT: digital Clock Drawing Test; sRCFT: simplified Rey-Osterrieth Complex Figure Test.

Furthermore, we used 4 metrics to measure the classification performance of the models, including accuracy, sensitivity, specificity, and area under the curve, as shown in Table 5. Receiver operating characteristic curves for logistic regression models were plotted, as depicted in Figure 5. Notably, the sRCFT delayed recall reached 88.4% (183/207) of the highest detection accuracy, while its specificity results were also the best (90/99, 91%). Meanwhile, the area under the curve indicating the authenticity of the detection method is the highest in sRCFT delayed recall, signifying superior performance. In the dCDT dual task, 88% (95/108) of all predicted patients with MCI were actual patients with MCI. In contrast, the sRCFT raw copy yielded lower classification results, with an accuracy of 82.1% (170/207). Our interpretation of these findings is that relatively complex tasks (eg, dCDT dual task and sRCFT delayed recall) are associated with integrated cognitive abilities, including executive functioning, language comprehension and expression, information extraction, and spatial visualization, making them more effective in detecting cognitive deficits.

**Table 5:** Diagnostic value of digital drawing and home dragging tasks in healthy older adults and patients with mild cognitive impairment.

Model	Accuracy	Sensitivity	Specificity	AUC^a^ (95% CI)	*P* value
dCDT^b^: single task	0.845	0.861	0.828	0.904 (0.846-0.962)	<.001
dCDT: dual task	0.855	0.880	0.828	0.892 (0.830-0.954)	<.001
sRCFT^c^: raw copy	0.816	0.843	0.788	0.885 (0.820-0.950)	<.001
sRCFT: delayed recall	0.884	0.861	0.909	0.945 (0.903-0.986)	<.001
clock: drag and drop	0.845	0.861	0.828	0.904 (0.846-0.962)	<.001
sRCFT: point and line	0.855	0.861	0.848	0.912 (0.859-0.965)	<.001

^a^AUC: area under the curve.

^b^dCDT: digital Clock Drawing Test.

^c^sRCFT: simplified Rey-Osterrieth Complex Figure Test.

### Comparison of Our Cognitive System With Existing Studies

We compared 4 aspects of sensitivity, specificity, administration time, and self-administration with digital cognitive tests identified in recent years to identify MCI and dementia, as shown in Table 6. For specificity and sensitivity, we averaged the results of the 4 drawing tasks and the 2 dragging tasks. The findings suggest that our cognitive system is equivalent to the best-performing Vigilance and Memory Test [45] in accurately discriminating patients with MCI. Similarly, in identifying healthy older adults, the performance on both types of tasks surpassed that observed in most recent studies, except for slightly lower performance than the screening system based on ROCF [46]. Although the total time for the digital drawing tasks was approximately 15 minutes, each task ranged from 3 to 5 minutes, aligning with the administration time (typically between <5 and 35 minutes) observed in most studies. Moreover, in the home dragging task, the time was generally shorter, and participants could achieve self-administration, a feature rarely observed in previous studies.

**Table 6:** Our cognitive system compared to existing mild cognitive impairment detection studies.

Test name	Study and year	Sensitivity	Specificity	Administration time (min)	Self- administration
Digital Tree Drawing Test	Robens et al [15], 2019	0.560	0.830	<5	—^a^
FACE^b^-memory	Alegret et al [41], 2020	0.734	0.721	30	Yes
Vigilance and Memory Test	Fung and Lam [45], 2020	0.861	0.753	15	No
Screening System based on ROCF^c^	Cheah et al [46], 2019	0.756	0.864	35	No
Digital Clock Drawing Test	Müller et al [47], 2019	0.854	0.775	<5	—
Smart Aging Serious Game	Cabinio et al [48], 2020	0.844	0.755	—	—
Virtual supermarket	Eraslan Boz et al [49], 2020	0.630	0.720	25	Yes
Digital drawing task (average)	This paper	0.861	0.838	15	No
Home dragging task (average)	This paper	0.861	0.838	<5	Yes

^a^Not applicable.

^b^FACE: Face-Name Associative Memory Exam.

^c^ROCF: Rey-Osterrieth Complex Figure.

### User Experience of Cognitive Screening Process

**Figure 6:**
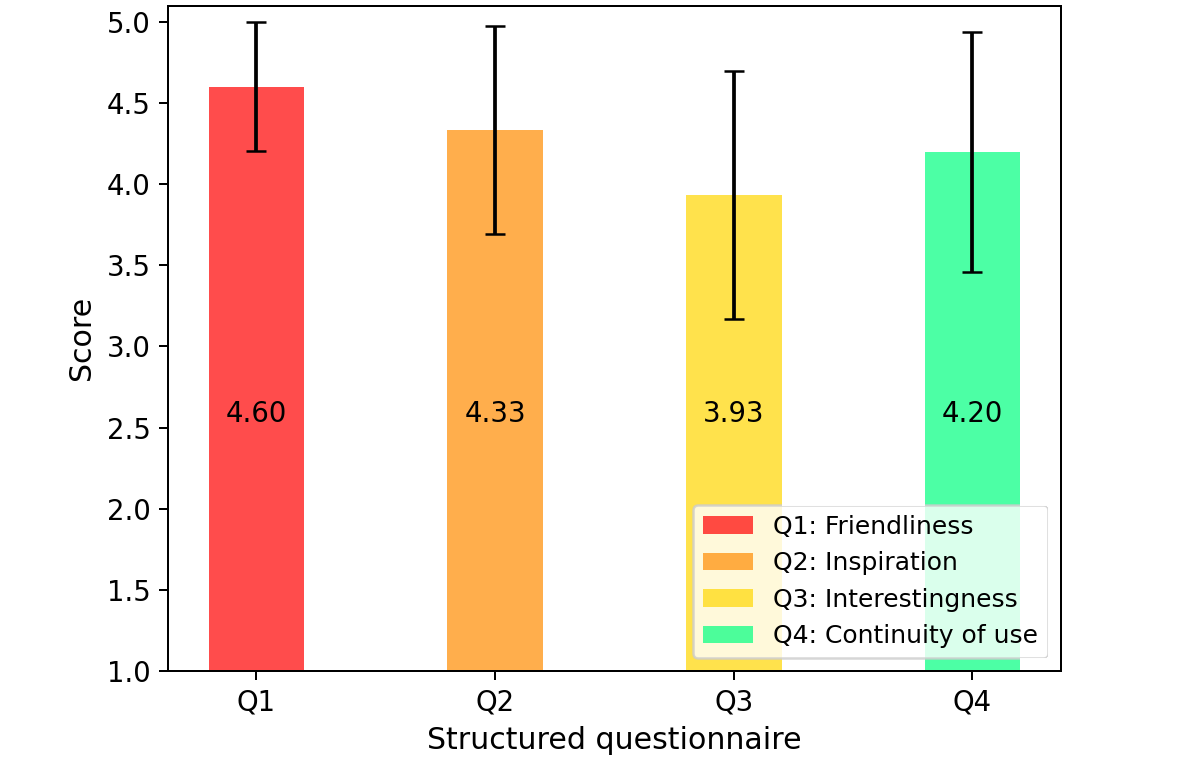
User experience results with the structure questionnaire. Q: question.

Figure 6 illustrates the results of the questionnaire experiment, where the error bars represent SD. All 207 participants rated the screening process highly across all aspects. The mean score for question 1 (4.60) was the highest among all questions, suggesting that our system was user-friendly, enabling most older adults to complete each test. However, the score for question 3 (3.93) was the lowest of all 4 questions, possibly due to the system’s limited interaction, resulting in tasks being perceived as less engaging. Furthermore, most older adults (179/207, 86.5%) reported that our system engaged their cognition and helped identify cognitive deficits during self-screening (question 2: 4.33), expressing an interest in continued use in the future (question 4: 4.20).

Similarly, we gleaned valuable insights from unstructured interviews. All participants concurred that the screening tool is user-friendly and practical. Furthermore, older adults offered valuable suggestions for enhancing the screening tool: (1) integrating social features to bolster user engagement and (2) providing instructional videos for each test to aid participants in comprehending the task requirements and procedures more effectively.

## Discussion

### Design Advantages Behind “Drawing and Dragging” Tasks

First, we digitized the traditional drawing test and assessed cognitive performance using a digital pen and tablet. The screening system recorded task-related data (eg, time, score, stroke, frequency, and sequence) and automatically uploaded these data to a remote cloud server where they could be analyzed and visualized through statistical analysis for easy access by neurologists and patients. Second, we captured the hand motor function features of each module (ie, clock face, number, and pointer) in dCDT to further explore the specific cognitive deficits of patients with MCI, which are rarely mentioned in the existing literature. In addition, the sRCFT was well received by older adults in practical testing settings and was easier to administer. Third, we introduced 3 tasks related to language function in the dCDT, expanding the assessment capabilities of the drawing system and increasing its potential application in detecting various cognitive impairments. Fourth, each task was time efficient, taking only 3 to 5 minutes. Older adults were willing to engage with and accept this, effectively avoiding the negative emotional impact of prolonged testing. Fifth, the screening system provides drawing and dragging interaction, which maintains the traditional drawing test requirements while meeting patients’ needs for self-perception screening. In other words, the system can be applied to hospitals and clinics to facilitate physicians to understand patients’ conditions in a timely and effective manner and can also meet the self-assessment of the cognitive level of older adults at home and provide an early warning means.

### Detection Accuracy and Interpretability of Features

As for the cognitive assessment results, the 4 drawing and 2 dragging tasks performed well in discriminating abilities (average accuracy of 85.2%), comparable to the results of alternative state-of-the-art methods [47,50,51]. In addition, statistical hypothesis testing was conducted on the 5 categories of critical features acquired during the cognitive assessment. The analysis results provide three insights: (1) time features are the most prominent in cognitive assessment; (2) key features vary in different tasks; (3) most older adults strive for perfection in drawing or dragging tasks and ignore the features of movement kinematics, such as long-term stagnation in the air, multiple drawings to make the figure symmetrical, and slow drawing or dragging speed.

Regarding the digital pen stroke data analysis, we compared differences between the 2 groups in the dCDT. Typically, healthy older adults prioritized drawing the numbers 12, 6, 3, and 9 to divide the clock face area accurately and make the clock more perfect, partly reflecting their ability to generate adequate decision-making strategies [52].

In contrast, patients with MCI were accustomed to rotating the tablet to write numbers in sequence, counterclockwise to write numbers from 12, unable to indicate “10 minutes past 11 o’clock” and refine the scale between numbers 11 and 12. These behavioral patterns reveal deficiencies in patients with MCI’s abilities related to time perception, numerical comprehension, and the organization and planning required for executing cognitive tasks [30,53]. On the other hand, when comparing drawing sequences between healthy older adults and patients with MCI in the sRCFT, most participants followed similar patterns: (1) from top to bottom, (2) from global to local, and (3) checking and filling in missing information. Some patients used unconventional methods, such as mirror drawing or completing the global structure last, possibly due to personal painting habits. Notably, patients with MCI scored lower and omitted many local features in delayed recall, suggesting a link between the loss of detailed features and visual-spatial dysfunction or deficits in visual-spatial working memory [54,55]. Furthermore, our study primarily focused on extracting local features, which may offer better identification of patients with MCI.

### Experience of “Drawing and Dragging” Tasks

In terms of user experience, 207 participants rated the ease of use positively, with an average score of 4.60 (SD 0.398). In contrast, the average score for task enjoyment was 3.93 (SD 0.765), which is less desirable. The “drawing and dragging” tasks are designed as a serious game to maintain rigor while assessing participants’ perceptions through teaching and training methods. However, this approach may be less conducive to generating enjoyment and may challenge user interest, leading to a perception of dullness and tedium. Moving forward, our focus will be on enhancing plot design and incentives and making it resemble a real game by incorporating interactivity and feedback mechanisms.

### Limitations and Future Work

For our study, it is important to acknowledge several potential limitations and proposed solutions. First, our sample size was relatively small, comprising only 207 participants. To address this, we intend to conduct a longitudinal study to validate the efficacy of the developed MCI screening tool, aiming to recruit a larger and more representative sample. Second, considering the visual condition of older adults, the “drawing and dragging” tasks were performed on a 10.2-inch touchscreen tablet. Therefore, whether this study’s experimental results can be extrapolated to smaller-screen smartphones remains to be determined. We will validate these smartphone tasks to fully evaluate their applicability, considering the user experience on different devices.

### Conclusions

We present an MCI detection system based on the digital “drawing and dragging” by assessing the movement kinetics of older adults during various tasks. The interactive system comprises 4 digital drawing tasks and 2 home dragging tasks. We report how these 6 cognitive tasks are designed, optimized, and evaluated through research on traditional clinical screening tools, discussions with related neurologists, and user experience feedback. Then, specific parameters were combined for different tasks, and different mixed models were constructed. The experimental outcomes demonstrate the efficacy of the proposed model in distinguishing patients with MCI from healthy older adults, garnering positive reception from multiple users. In a broader context, this study advances novel approaches for nuanced feature extraction of movement kinetics in individuals with MCI, offering tangible support for developing cognitive impairment detection systems.

## Appendix

Multimedia Appendix 1 Experiment introduction video.

Multimedia Appendix 2 Detailed description of features.

Multimedia Appendix 3 Visualization of drawing sequences.

## Abbreviations

AD: Alzheimer dementia
CDR: Clinical Dementia Rating
CDT: Clock Drawing Test
dCDT: digital Clock Drawing Test
HC: healthy control
MCI: mild cognitive impairment
MMSE: Mini-Mental State Examination
MoCA: Montreal Cognitive Assessment
RCFT: Rey-Osterrieth Complex Figure Test
ROCF: Rey-Osterrieth Complex Figure
sRCFT: simplified Rey-Osterrieth Complex Figure Test
