# Supplementary material for: Detection of Mild Cognitive Impairment Through Hand Motor Function Under Digital Cognitive Test: Mixed Methods Study
Source: JMIR Mhealth Uhealth. 2024 Jun 26;12:e48777. doi: 10.2196/48777 (PMC11237787; doi:10.2196/48777)
Supplement: Multimedia Appendix 2 [file mhealth_v12i1e48777_app2.pdf]

Detailed description of features

We extracted five types of features related to hand motor function and investigated whether they can capture mild cognitive impairment. These features include time, stroke, frequency, score, and sequence. All these features were extracted from the drawing or dragging tasks participated by older adults. The details of these five types of features are described below.

- Time-based feature: Sluggish reaction times are commonly observed in patients with cognitive impairment. Thus, we calculated the time of the first stroke (the time from the beginning to the first stroke), the time in air (i.e., the time the pen stay in the air), the time on surface (i.e., the time the pen touches the tablet) and the time being dragged (numbers and pointers completion times respectively and total drag time).
- Stroke-based feature: Pen stroke is the trace of motion recorded from the moment the pen touches the screen to the moment the pen leaves. To analyze stroke behaviors, we evaluated the total stroke length and pen-up stroke length (i.e., in-air distance while moving the pen from one stroke to the next).
- Frequency-based feature: Drawing frequency was defined as the number of touches between the digital pen and the writing tablet. It was mainly calculated by accumulating the number of times the digital pen moved from the air to the writing board each time. Similarly, the drag frequency is calculated by the number of times between the hand and the drag component (i.e., number, pointer).
- Score-based feature: Drawing quality will intuitively reflect the cognitive ability of the participants. In digital drawing tasks, participants' scores were given by the experimenter clicking a scoring button after each task. In home dragging tasks, Figure 1 (a) shows the scoring elements of the clock “drag & drop” task, where the “red box” represents the position and order of the numbers “1-12” (e.g., blue number 6 in the correct position), and the yellow sector and green sector represent the scale range required for the hour pointer and minute pointer respectively. In order to solve the problem that the result of the participant's dragged number may not wholly coincide with our design position, we stipulate that as long as the dragged number intersects with our design position, it will be assigned the designed position value. Figure 1 (b) shows the sRCFT “point & line” task scoring key points, where the “green box” represents the drag position of a circle with three dots. All remaining scores are judged by counting the number of lines one vertex connects to other vertices.
- Sequence-based features: The participants' organizational skills were assessed by analyzing the stroke sequences. Specifically, participants' total number of strokes was divided into segments according to stroke sequence, and each segment was coded with a series of colors ranging from red to purple. For example, when a participant drew a total of 20 strokes, the first 4 strokes accounting for the first 20% (4/20) were designated as red, the next 20% (4/20) were selected as orange, and so on.

Besides extracting features covering the global process, we investigated some local modules and their features to detect MCI patients from healthy individuals more accurately and to guide clinical applications. In particular, it is about the clock face, number and pointer modules in dCDT. Figure 1 (c) shows the detection details for each module of the clock drawing test, with one participant drawing the clock face in four strokes following the sequence of red, yellow, blue, and green. Firstly, according to the definition, the center and radius of the clock face (treated as a circle) can be determined by any three points on the plane. Thus, we compared the stroke sequences of dCDT and

divided the longest stroke (i.e., a yellow segment in Figure 1(c)) into four equal parts to obtain three coordinates, thereby establishing the center and radius. We define the drawing process in dCDT, where stroke sequences are smaller than the longest stroke sequence as the clock face module. Simultaneously, we also calculated the distance from the starting point coordinates of five strokes after the longest stroke to the center, relative to the radius, to determine if these strokes were perfecting the clock face (e.g., the yellow and green segments in Figure 1(c)). Furthermore, following the advice of neurologists, we defined the area covering 40% of the clock face radius as the pointer region. A stroke passing through the pointer region indicates the participant is currently in the pointer module; otherwise, it is considered drawing numbers.

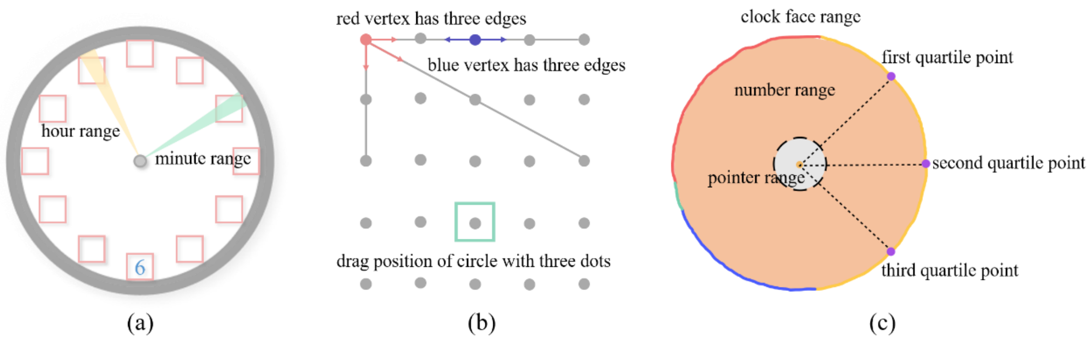

Figure 1. Feature details for home dragging tasks. (a) Scoring points for the clock “drag & drop” task; (b) Scoring points for the sRCFT “point & line” task; (c) Detection details of each module of the clock drawing test.
