# Supplementary material for: Detection of Mild Cognitive Impairment Through Hand Motor Function Under Digital Cognitive Test: Mixed Methods Study
Source: JMIR Mhealth Uhealth. 2024 Jun 26;12:e48777. doi: 10.2196/48777 (PMC11237787; doi:10.2196/48777)
Supplement: Multimedia Appendix 3 [file mhealth_v12i1e48777_app3.pdf]

Visualization of drawing sequences

In the digital drawing tasks, the figure drawn by participants were converted into pseudo-color images consisting of a series of color-coded stroke sequences, as shown below.

A

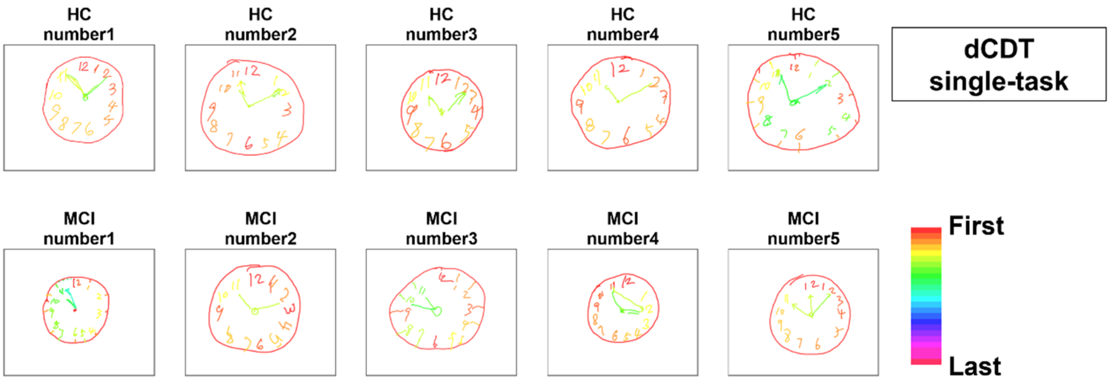

B

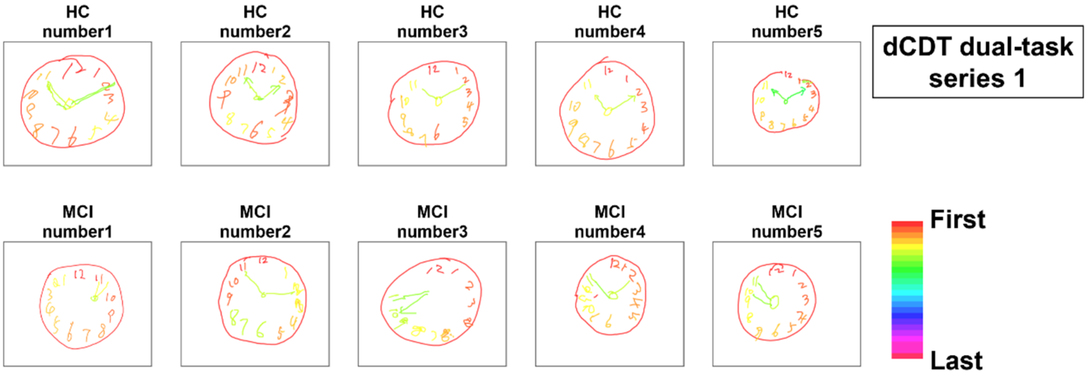

C

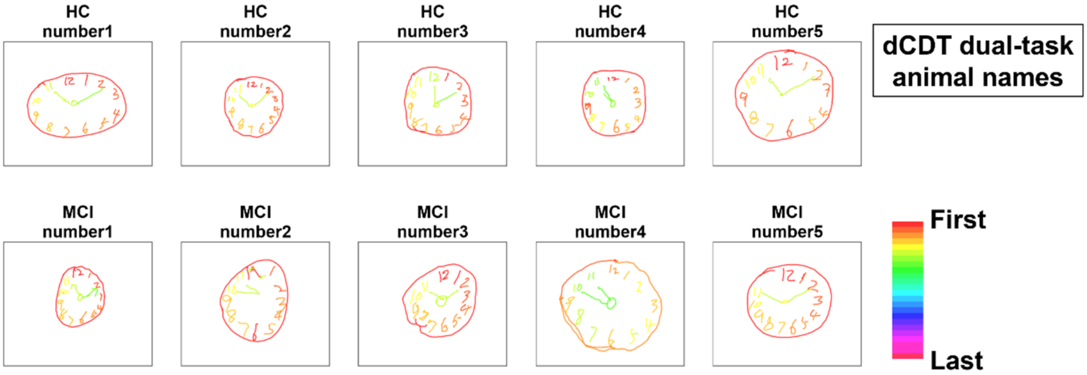

D

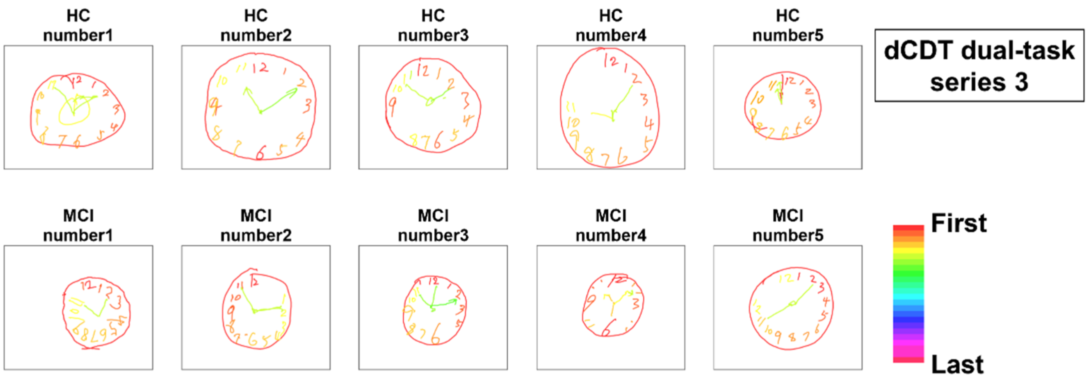
HC number1
HC number2
HC number3
HC number4
HC number5
dCDT dual-task series 3
MCI number1
MCI number2
MCI number3
MCI number4
MCI number5
First
Last

E

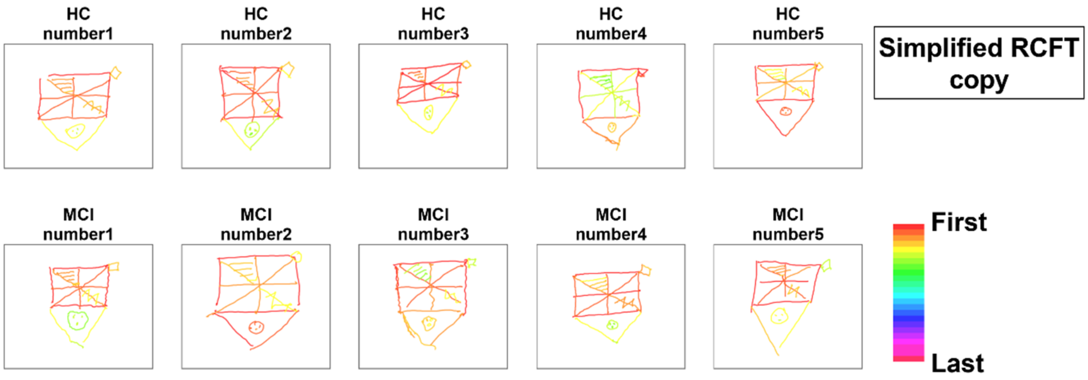
HC number1
HC number2
HC number3
HC number4
HC number5
Simplified RCFT copy
MCI number1
MCI number2
MCI number3
MCI number4
MCI number5
First
Last

F

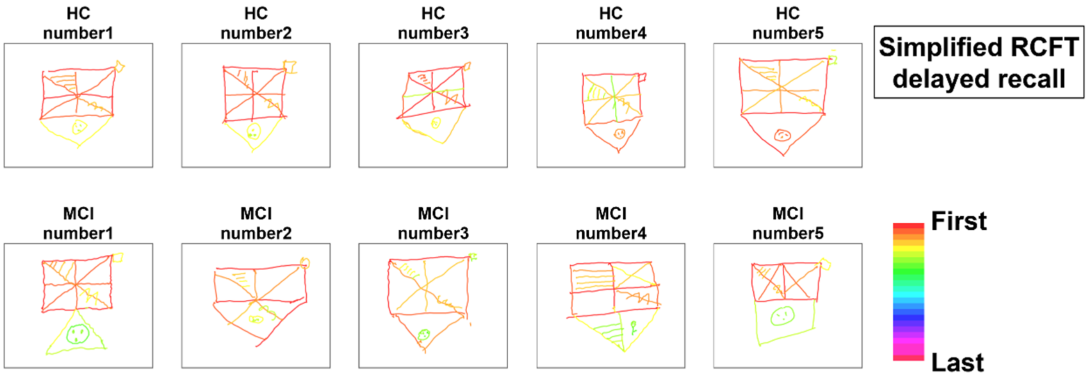
HC number1
HC number2
HC number3
HC number4
HC number5
Simplified RCFT delayed recall
MCI number1
MCI number2
MCI number3
MCI number4
MCI number5
First
Last
